# Supplementary material for: Effectiveness of Live Attenuated Varicella-Zoster Vaccine in Adults Older than 50 Years in Japan: A Retrospective Cohort Study
Source: Vaccines (Basel). 2023 Jan 25;11(2):259. doi: 10.3390/vaccines11020259 (PMC9958742; doi:10.3390/vaccines11020259)
Supplement: Supplementary file 1 [file vaccines-11-00259-s001.zip › vaccines-2086105-supplementary.pdf]

Table S1. Information from medical institutions.

Outcome	Vaccinated Group ($n = 16$)	Unvaccinated Group ($n = 10$)	p- value [a]
HZ diagnosis	13 (81)	9 (90)	1.0
Rapid examination	16 (100)	10 (100)	0.38
Sequala	2 (13)	4 (40)	0.16
Antiviral treatment	12 (75)	8 (80)	1.0
Analgesia	9 (56)	8 (80)	0.40

[a] Chi-square test or Fisher's exact test were used as appropriate; HZ: Herpes zoster

Table S2. The severity and duration of pain in both groups.

Severity/Duration	HZ Patients in Vaccinated Group ($n = 26$)	HZ Patients in Unvaccinated Group ($n = 22$)	p- value [a]
Mild	3 (11)	7 (32)	
Moderate	16 (61)	11 (50)	0.97
Severe	7 (27)	4 (18)	
Short (less than 2 weeks)	16 (61)	16 (73)	
Moderate (2 weeks ~ a month)	8 (31)	5 (23)	0.73
Long (more than a month)	2 (8)	1 (4)	

[a] Chi-square test or Fisher's exact test were used as appropriate.
